# Visuospatial Working Memory Mediates the Relationship Between Executive Functioning and Spatial Ability

**DOI:** 10.3389/fpsyg.2018.02302

**Published:** 2018-12-14

**Authors:** Lu Wang, Jocelyn Bolin, Zhenqiu Lu, Martha Carr

**Affiliations:** ^1^Department of Educational Psychology, Ball State University, Muncie, IN, United States; ^2^Department of Educational Psychology, University of Georgia, Athens, GA, United States

**Keywords:** executive functioning (EF), visuospatial working memory (VSWM), verbal working memory (VWM), mental rotation, dominance analysis, mediation analysis

## Abstract

This study investigates the relationships among EF, VSWM, VWM, and spatial ability (mental rotation) at the construct level through testing a series of mediation models. A second objective of the study is to investigate whether the mediation relationship changes depending on the secondary demand of the tests used to measure EF. Covariates age and gender were controlled for in theses analyses. The results showed that when the Tower test, an EF test with a spatial secondary demand, was used to represent EF, VSWM significantly mediated the relationship between EF and mental rotation. However, when the composite inhibition and switching scores from the Color-Interference Test, an EF test with a verbal secondary demand, was used to represent EF, VSWM no longer significantly mediated the relationship between EF and mental rotation. This pattern of findings suggests that the test effect is real. Therefore, a grain of salt should be taken when interpreting prior findings concerning the relationship between EF and VSWM, when EF was measured using a variety of instruments, some of which have a spatial secondary demand, whereas others do not. Regarding VWM as a mediator, it was not found to be significantly mediating the relationship between EF and mental rotation, regardless of whether the Tower test or the Color-Interference Test was used to measure EF. A third objective of the study is to investigate the relative importance of EF, VSWM, and VWM in predicting mental rotation via dominance analysis. The results showed that VSWM is more important than VWM in explaining individual differences in mental rotation; the Tower test is more important than the Color-Interference Test in explaining individual differences in mental rotation. These findings again suggest that cautions need to be taken when interpreting prior findings that showed EF is highly involved in spatial ability, as test effect is real and may at least be partially be responsible for the linkage between the two constructs.

## Introduction

In this study, we explored the interrelationships among visuospatial working memory (VSWM), verbal working memory (VWM), executive functioning (EF), as well as the extent these cognitive factors contribute to individual differences in spatial ability, an often neglected higher-order thinking skill until recent years. In addition, we probed the question of the relative contributions of EF, VSWM, and VWM in predicting individual differences in spatial ability. Many existing studies documented the vital roles played by various components of working memory in explaining individual differences in higher-order thinking skills, such as reading comprehension, logical reasoning, and problem solving (Miyake and Shah, 1999; Conway et al., 2005). While multiple working memory models were developed over the years, which embody different empirical and theoretical orientations, e.g., computer based, multi-componential, storage-and-processing, generic, to name just a few (for more information, see a recent review by Cowan, 2017), this study endorses a multi-componential view of working memory that partitions working memory into a visuospatial, a verbal, and an EF components (see Baddeley, 2012). This is because this view of working memory was widely endorsed in prior research that investigated individual differences in higher-order thinking skills.

In the research literature, VSWM and VWM highlight the distinction between the content of the materials being stored and processed. VSWM is where 2D and 3D representations are temporarily stored while being simultaneously processed. VWM is where linguistic and numerical information is temporarily stored while being simultaneously processed.

Executive functioning encompasses a range of related cognitive functions (see Miyake et al., 2000; Bull and Scerif, 2001; Lehto et al., 2003; St Clair-Thompson and Gathercole, 2006; Swanson, 2006; Bull et al., 2008). A common way to partition the EF is by dividing it into an inhibition, the ability to inhibit task-irrelevant information or predominant responses, a switching/cognitive flexibility component, the ability to shift attention among multiple tasks, and an updating component, the ability to constantly refresh information temporarily stored in working memory sensory subsystems (see Miyake et al., 2000). The updating component of the EF overlaps with working memory sensory subsystems in that it carries out the task of temporarily holding incoming information while simultaneously updating new information as it is picked up by the sensory subsystems.

Spatial ability refers to the ability to represent, transform, generate, and recall symbolic information (Linn and Petersen, 1985; Lohman, 1996). Mental rotation is a type of spatial ability that has been extensively studied individual differences research. Solving mental rotation problems typically requires quickly rotating two or three-dimensional objects in mind. In the following sections, we review studies that investigated the relationship between VSWM and mental rotation, between VWM and mental rotation, and between EF and mental rotation.

### Visuospatial Working Memory and Spatial Ability

In prior studies, VSWM has been measured by complex span tasks with either a combined passive visuospatial storage and an active visuospatial processing demand, as in the case of rotation-arrow span (see Shah and Miyake, 1996), or a combined passive visuospatial storage and an active verbal processing demand, as in the case of verification-arrow span (see Shah and Miyake, 1996). Forward and backward spatial span (Wechsler, 1997, also known as the Corsi blocks tapping task), is another commonly used measure of VSWM that has a primary visuospatial storage demand and an active secondary processing demand that involves EF, as in the case of mentally reversing the order of the tapped patterns. There is also some evidence from behavioral studies that indicated that VSWM and spatial ability are closely related constructs (see Shah and Miyake, 1996; Reuhkala, 2001; Kaufman, 2007; Kyttälä and Lehto, 2008).

Neuroimaging research further illustrated the close relationship between VSWM and spatial ability by revealing similar brain activities when participants performed VSWM and spatial ability (mental rotation) tasks (see Levin et al., 2005), respectively. In addition, an EEG study by Christie et al. (2013) showed that scores on a mental rotation test mediated gender differences in performance on a computerized VSWM task and at least in males, mental rotation completely mediated the correlation between VSWM task performance and the amplitude of P300, a marker of activities in VSWM.

Cornoldi and Vecchi (2004) advanced a theory that attributed individual differences in spatial ability to two sources of variances. One source is attributed to the ability to temporarily store mental images. The second source is attributed to the ability to actively process or manipulate mental images. These two sources of variances are aligned in cognitive demand with the two components of VSWM described previously. In a nutshell, evidence from multiple sources indicates that VSWM and spatial ability are distinct but closely related constructs.

### Verbal Working Memory and Spatial Ability

Verbal working memory is involved in disambiguating, parsing, and integrating textual information during reading comprehension (Daneman and Carpenter, 1980). Similar to its counterpart VSWM, VWM also has a passive storage component and an active processing component. VWM capacity reflects the ability to accurately retain the identities and serial orders of verbal or numerical strings. A typical verbal memory span is about six or seven digits (Baddeley, 2012). Commonly used VWM tasks are forward and backward digit span and complex span tasks featuring a primary verbal storage demand and a secondary active processing demand of domain-general (e.g., operation span task, Turner and Engle, 1989; random number generation task, Baddeley, 1996), verbal (e.g., reading span task, Daneman and Carpenter, 1980; verification-word span, Shah and Miyake, 1996), or visuospatial natures (e.g., rotation-word span, Shah and Miyake, 1996).

Studies investigating the relationship between VWM and spatial ability yield mixed findings. On the one hand, Pardo-Vazquez and Fernandez-Rey (2012) showed that a VWM test with a combined passive verbal storage and an active domain-general processing demand significantly predicted individual differences in spatial ability, as measured by mental rotation tests. Similarly, Kaufman (2007) found that when VSWM and VWM were simultaneously in the model as predictors of spatial ability (mental rotation) in a sample comprised of high-achieving college students age ranged from16 to18, VWM significantly predicted performance on two mental rotation tests. The magnitude of the predictive relationship was stronger between VSWM and mental rotation than between VWM and spatial ability, however. On the other hand, Shah and Miyake (1996) found that while VWM, as measured by a complex verbal span task, significantly correlated with verbal ability measures, it did not correlate with spatial ability measures (Experiment 1). The pattern of findings reported in Experiment 1 was replicated in Experiment 2 that used a selective interference paradigm. Similarly, Christie et al. (2013) study also did not find VWM to be significantly predicting spatial ability (mental rotation) in their male participants.

### Executive Functioning and Spatial Ability

There is some evidence that EF is implicated in spatial ability. In a study involving healthy young adults that investigated the factor structure and the interrelationships among EF, visuospatial short-term memory, VSWM, and three types of spatial ability factors identified by Linn and Petersen (1985) and Miyake et al. (2001) found that EF is strongly implicated in spatial relations (mental rotation) and spatial visualization. The latter requires multi-step mental rotation. Additionally, the same study also showed that visuospatial short-term memory and VSWM were moderately related to EF. In a similar vein, St Clair-Thompson and Gathercole (2006) study found that the EF predicted both VWM and VSWM. Based on this pattern of findings, as well as studies showing spatial ability (especially mental rotation) and VSWM are closely related constructs, it is possible that EF’s impact on spatial ability may at least be partially mediated by VWM, and possibly completely mediated by VSWM.

### Rationale

The present study is built upon Miyake et al. (2001) and Kaufman (2007) studies. It sought to understand the relationships among EF, VSWM, VWM, and spatial ability (mental rotation) at the construct level through testing a series of mediation models—with VSWM or VWM serving as the sole mediator or with both VSWM and VWM serving as the mediators between EF and spatial ability (mental rotation). In addition, the present study explores the test effect concerning two commonly used EF measures, one of which has a spatial secondary demand (the Tower Test) and the other of which has a verbal secondary demand (the Color-Interference Test), on the mediation relationships among EF, VSWM, VWM, and spatial ability. To understand the unique mediation effects of VSWM and VWM, as well as their conjoint mediation effects, three separate mediation models were tested (i.e., Models 1, 2, and 3). To investigate whether the significance of the mediation relationship varies, depending on whether the Tower Test or the Color-Interference Test was used to measure EF, two separate mediation models with each test serving as the indicator of the EF construct were tested (i.e., Models a and b). The crossing over of Models 1, 2, and 3 with Models a and b resulted in six models—i.e., Models 1a, 1b, 2a, 2b, 3a, and 3b. Finally, to determine the relative importance of EF, VSWM, and VWM in predicting mental rotation, dominance analysis was performed. Covariates age and gender were controlled for in both the mediation analyses and the dominance analyses.

## Materials and Methods

### Participants

Participants were 144 (38 males) adults attending a medium-sized public research university in the Southeast region of the United States. The ethnic composition of the study sample is comprised of 79 percent of European Americans, 5.6 percent of African Americans, 4.2 percent of Asian Americans, 4.2 percent of Hispanic Americans, and 6.3 percent of participants who identified themselves as “Other.” The ethnic composition of the study sample is similar to that of the geographical area where the data was collected. The average age of the research participants was 22.51 years (*SD* = 5.58 years). The research participants’ college majors encompassed social sciences (73.6 percent), natural sciences (13.9 percent), and humanities (12.5 percent). The participants were recruited either by flyers that were put up campus-wide or through course instructors. The study was approved by the university’s Institutional Review Board (IRB).

### Procedures

Participants were individually tested in a quiet conference room. A battery of tests, including Forward and Backward Digit Span (Wechsler, 1997), Forward and Backward Spatial Span (Wechsler, 1997), Color-Interference Test (Delis, 2001), Tower Test (Delis, 2001), and Mental Rotation Test (Vandenberg and Kuse, 1978) were administered with the order of the tests counterbalanced. All tests were administered following a standardized procedure specified in the test manuals. The average time for each participant to complete the full battery was approximately an hour. A demographic survey was administered at the end of each testing session.

### Materials

#### Forward and Backward Digit Span Tests (Wechsler, 1997)^1^

Forward and Backward Digit Span tests were used to measure VWM. Task stimuli are numerical strings (from 1 to 9). The length of the numerical strings increases incrementally from the one item to the next. There are eight items on the Forward Digit Span and seven items on the Backward Digit Span. Each item has two trials that are numerical strings of the same length. The numerical strings were read at the rate of one digit per second. Participants score one point for having correctly reproduced the serial order of each numerical sequence. The task discontinues if participants had two successive failures in reproducing the serial order of the numerical sequence. The task stimuli and administration procedures of Backward Digit Span test are similar to Forward Digit Span, except that participants were instructed to repeat the digit sequence in the reverse order of what they hear. The total score of Forward and Backward Digit Span was used in the ensuing analyses.

#### Forward and Backward Spatial Span Tests (Wechsler, 1997)^2^

Forward and Backward Spatial Span tests were used to measure VSWM. The task stimuli and the basic administration procedures of Forward and Backward Spatial Span are similar to the Corsi blocks-tapping task (Milner, 1971). Nine blocks are attached to a flat base and the participants were instructed to tap the blocks in the same (forward spatial span) or reverse (backward spatial span) order as presented. The block sequences were tapped at the rate of one block per second. There are eight items on the Forward Spatial Span and seven items on the Backward Spatial Span. Each item has two trials that are block sequences of the same length. The length of the block sequence tapped increases incrementally from one item to the next. Participants score one point for correctly reproducing each block sequence. The task discontinues if participants had two successive failures in reproducing the serial order of the block sequence. The task stimuli and administration procedures of Backward Spatial Span test are similar to Forward Spatial Span, except that participants were instructed to tap the block sequence in the reverse order of what they saw. The total score of Forward and Backward Spatial Span was used in the ensuing analyses.

#### Color-Interference Test (Delis, 2001)^3^

The Color-Interference Test (hereafter, the CIT) was one of the two measures of EF used in this study that has a strong verbal secondary demand. It is taken from the Delis–Kaplan Executive Functioning System (D-KEFS) test booklet. The task stimuli are ink blocks printed in colors (condition 1), color words printed in colors that are consistent with the words’ semantic meanings (condition 2), color words printed in colors that are inconsistent with the words’ semantic meanings (conditions 3 and 4). Conditions 1 and 2 are the baseline conditions. In condition 3, participants were instructed to name the ink colors of the printed words. In condition 4, participants were instructed to switch between reading the printed words (when those words are enclosed by squares) and naming the ink colors in which the words are printed (when the words are not enclosed). Because the task stimuli presented in the four conditions of the CIT entail either assigning verbal labels to printed colors (i.e., naming the colors of the ink blocks) or decoding the semantic meanings of the printed words (i.e., reading the printed words), the CIT can be said to have a strong verbal secondary demand. As reviewed previously, inhibition and switching are two important components of the EF (see Miyake et al., 2000). Both components of the EF are reflected in the CITSW, a composite score that we derived, based on participants’ performance in the inhibition and switching conditions of the CIT. This composite score is used in the ensuing analyses as an indicator of EF.

#### Tower Test (Delis, 2001)^4^

The Tower Test was the other measure of EF used in this study. Unlike the CIT, the Tower test has a strong visuospatial secondary demand. The apparatus is comprised of a solid wooden base with three wooden pegs and five moveable ring disks of various sizes. Participants were instructed to construct the designs provided in the stimulus book within the allotted time (which varies for each item, depending on the item’s complexity) by moving around the ring disks. Participants were instructed to follow two rules: Never move more than one disk at a time and never place a larger disk on top of a smaller one. The total achievement score on this test is determined by the number of moves taken to complete each item within the allotted time. Fewer moves are associated with higher total achievement score. The Tower test total achievement score reflects the inhibition component of the EF through the ability to inhibit perseverative responding and the updating component of the EF through the ability to establish and maintain instructional set. The total achievement score on this test is used as the other indicator of EF in the ensuing analyses. Together with the CIT composite score, the Tower total achievement score captures all three components of the EF identified in Miyake et al. (2000). Because the primary goal of the study was to understand the interrelationships among EF, VSWM, VWM, and spatial ability at the construct level, we did not isolate each component of the EF in our analyses, although exploring EF’s on spatial ability by its components may be a logical next-step in this research program, as discussed in greater detail in the Section “Discussion.”

#### Vandenberg-Kuse Mental Rotation Test^5^

The Vandenberg-Kuse Mental Rotation Test (hereafter, the MRT, Vandenberg and Kuse, 1978) was used in this study to measure spatial ability. The test contains 20 items. The task stimuli are line drawings of block stimuli. The participants were required to match two of the four choices to a target figure displayed on the left. Incorrect choices were mirror images of the target or alternative block configurations. The test was administered with a 7-min limit, as recommended by the test developers. Following Vandenberg and Kuse (1978) and Peters et al. (1995) scoring guidelines, the participants received one point only if both correct answers were selected. The total score on this test is the sum of the points received on each item. This variable is used in the ensuing analysis.

#### Demographic Survey

A demographic survey was administered at the end of the study. The survey asked the participants to self-report demographic information concerning gender, age, and ethnicity. Gender and age were used as covariates in the mediation analyses.

### Data Analysis

#### Description of the Dataset

First, descriptive statistical analyses were run to gain a general understanding of the dataset. To better understand how performance on Digit Span, Spatial Span, Color-Interference Test (CIT), Tower Test, and Mental Rotation Test relate to one another, correlation analyses were performed. Specifically, the correlational analyses concern the relationships among the following variables of interest, CITINSW (i.e., scores on the combined inhibition and switching conditions of the Color-Interference Test, the first test of EF used in this study), TOWERA (i.e., the total achievement score on the Tower test, the second test of EF used in this study), DIGIT (i.e., the total score on digit span, test of VWM), CORSIT (i.e., the total score on spatial span, test of VSWM), MRTT (i.e., the total score on the Mental Rotation Test), and the two covariates, gender and age, respectively. For cases with missing values, all possible observed values were used for calculation without deletion of the full cases.

#### Dominance Analysis

To investigate the relative importance of predictors (Gender, Age, TOWERA, CITINSW, VSWM, and VWM) in predicting the dependant varaible, MRTT, dominance analysis was performed (Budescu, 1993; Azen and Budescu, 2003). Dominance analysis is a procedure that examines the *R*^2^ values for subset models with all possible combinations of predictors in multiple regression models. This approach in analyzing the relative contribution of different predictors to the outcome variable is intuitive, meaningful, and informative. The results can shed light on a variety of research questions pertaining to predictor importance.

#### Mediation Analysis

**FIGURE 1:**
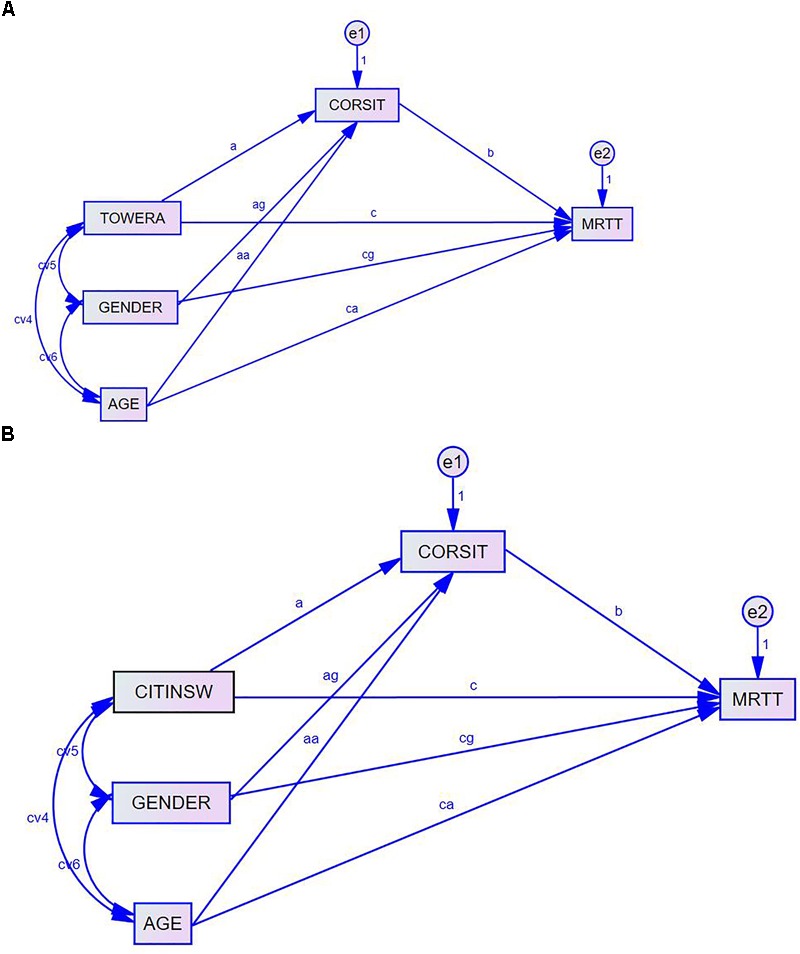
Path diagrams of the mediation analysis depicting how the relation between EF and mental rotation is mediated by VSWM (CORSIT) while controlling for age and gender. **(A)** Model 1.a for testing VSWM mediation from EF_tower (TOWERA). **(B)** Model 1.b for testing VSWM mediation from EF_color (CITINSW).

**FIGURE 2:**
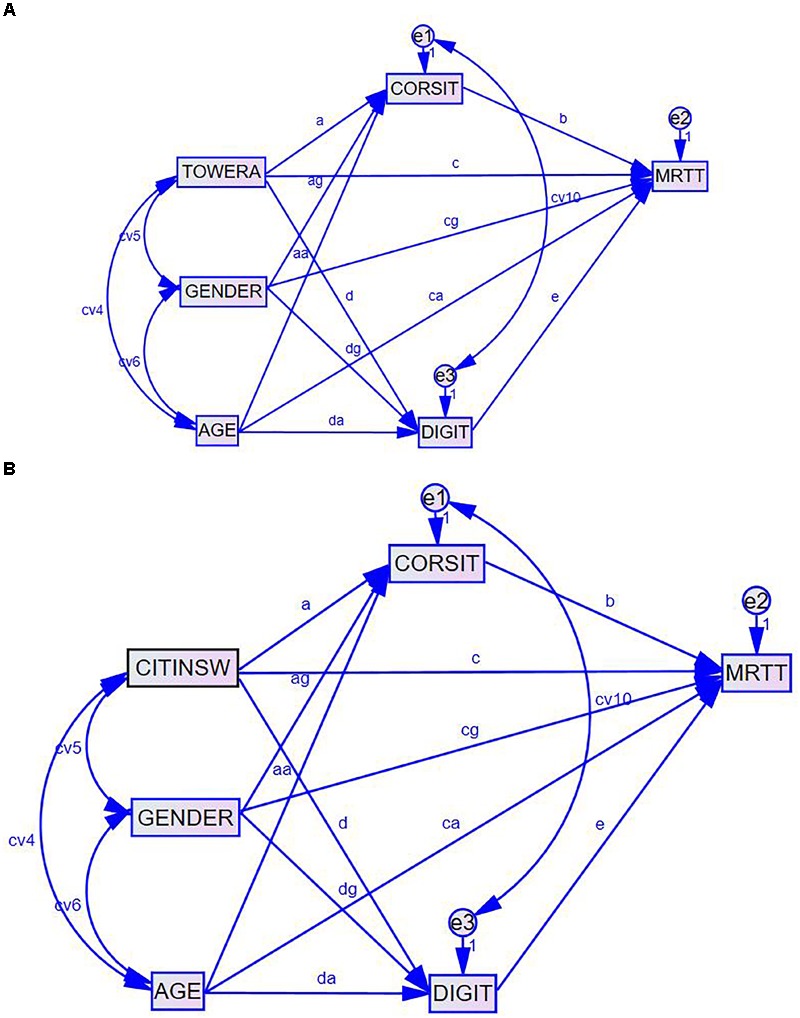
Path diagrams of the mediation analysis depicting how the relation between EF and mental rotation is mediated by VSWM (CORSIT) and VWM (DIGIT) while controlling for age and gender. **(A)** Model 3.a for testing VSWM and VWM mediation from EF_tower (TOWERA). **(B)** Model 3.b for testing VSWM and VWM mediation from EF_color (CITINSW).

To investigate the specific mediation effects of different components of working memory between EF and mental rotation, a series of mediation analyses were performed. Specifically, three mediation models were tested. Model 1 tested the mediation relationship of VSWM between EF and mental rotation (see Figure 1). Model 2 tested the mediation relationship of VWM between EF and mental rotation. Model 3 tested how VSWM and VWM simultaneously mediated the relationship between EF and mental rotation (see Figure 2). In all three mediation models, the total score of mental rotation served as the outcome variable. Two covariates, gender and age, were included in these models to control for their confounding effects. These analyses were based on the pre-established mediation procedures (Baron and Kenny, 1986). To obtain standard errors, Sobel tests (Sobel, 1982, 1986) were performed. The estimation method of Maximum Likelihood (ML) was used for these analyses such that all available information from the dataset was utilized in estimating the relevant coefficients. Although many software that have built-in Structural Equation Modeling-related procedures such as R, Mplus, EQS, Amos and OpenMx can be used to perform mediation analyses, AMOS path diagrams and analyses were evoked here due to the program’s user-friendliness and the ease of interpreting the program’s outputs. The results obtained from the other programs mentioned before yielded very similar results with the ones obtained through AMOS.

In the first set of mediation analyses, the statistical significance and the magnitude of the mediation effect (or indirect effects) via the mediator VSWM (i.e., CORSIT variable) were investigated (see Figures 1A,B for illustrations of the path diagrams of the two sub-models). One sub-model (Model 1.a) used the total achievement scores on the Tower test as the indicator of EF (see Figure 1A). The other sub-model (Model 1.b) used the composite scores on the inhibition and switching conditions of the Color-Interference Test as the indicator of EF (see Figure 1B). These mediation analyses investigated whether the indirect effect of the EF Tower test scores (Model 1.a) or the EF Color-Interference Test scores (Model 1.b) on mental rotation spatial ability via VSWM (CORSIT) was statistically significant as well as the magnitude of this indirect effect.

In the second set of mediation analyses, the statistical significance and the magnitude of the mediation effect (or indirect effects) via the mediator VWM (i.e., DIGIT variable) were investigated. Again, one sub-model (Model 2.a) used the total achievement scores on the Tower test as the indicator of EF. The other sub-model (Model 2.b.) used the composite scores on the inhibition and switching conditions of the Color-Interference Test as the indicator of EF. These mediation analyses investigated whether the indirect effect of the EF Tower test scores (Model 2.a) or the EF Color-Interference Test scores (Model 2.b) on mental rotation spatial ability via VWM (DIGIT) was statistically significant as well as the magnitude of this indirect effect.

In the third set of mediation models, the two mediators, VSWM and VWM, appeared in the same model such that their mediation effects were tested simultaneously. Based on the results from prior studies (e.g., Miyake et al., 2001; Kaufman, 2007), VSWM and VWM were treated as correlated constructs in this analysis. Figures 2A,B illustrated the path diagrams of the two sub-models involving different indicators of EF mentioned before.

## Results

### Descriptive Statistics

The descriptive statistics of the observed variables are displayed in Tables 1–3. Table 1 displays the means and the standard deviations (SD). Table 2 displays the variance-covariance matrix. Table 3 displays the Pearson correlations among all variables of interest. To calculate the variance-covariance matrix and the Pearson correlations for cases with missing values, pairwise deletion method was implemented.

**Table 1:** Descriptive statistics of the observed variables.

	GENDER	AGE	DIGIT	CORSIT	CITINSW	TOWERA	MRTT
Mean	0.264	22.632	10.528	10.854	11.092	10.042	7.958
*SD*	0.442	5.630	2.539	2.244	2.300	2.138	3.938

*Pairwise deletion method was implemented to compute the relevant statistics for cases with missing values.*

**Table 2:** Variance-covariance matrix of the observed variables.

	GENDER	AGE	DIGIT	CORSIT	CITINSW	TOWERA	MRTT
GENDER	**0.195**	0.664	0.146	0.080	-0.009	0.184	0.458
AGE	0.664	**31.702**	1.950	-0.284	-0.203	1.910	-0.994
DIGIT	0.146	1.950	**6.446**	1.581	0.405	1.180	1.162
CORSIT	0.080	-0.284	1.581	**5.034**	0.652	1.355	2.861
CITINSW	-0.009	-0.203	0.405	0.652	**5.289**	0.049	0.265
TOWERA	0.184	1.910	1.180	1.355	0.049	**4.571**	1.736
MRTT	0.458	-0.994	1.162	2.861	0.265	1.736	**15.508**

*Pairwise deletion method was implemented to compute the relevant statistics for cases with missing values.*

**Table 3:** Pearson correlation coefficients matrix of the observed variables.

	GENDER	AGE	DIGIT	CORSIT	CITINSW	TOWERA	MRTT
GENDER	1	0.266	0.130	0.081	-0.009	0.195	0.263
AGE	0.266	1	0.136	-0.022	-0.015	0.158	-0.044
DIGIT	0.130	0.136	1	0.277	0.069	0.217	0.116
CORSIT	0.081	-0.022	0.277	1	0.126	0.282	0.323
CITINSW	-0.009	-0.015	0.069	0.126	1	0.010	0.029
TOWERA	0.195	0.158	0.217	0.282	0.010	1	0.206
MRTT	0.263	-0.044	0.116	0.323	0.029	0.206	1

*The correlation coefficients were calculated with pair-wise deletion instead of list-wise deletion for the cases with missing values.*

### Mediation by VSWM

The first set of models looked at whether the prediction of mental rotation by EF was mediated by VSWM (CORSIT). In Model 1.a, EF was measured by the Tower test. In Model 1.b, EF was measured by the Color-Interference Test. Indirect effects were tested using the Sobel test (Sobel, 1982, 1986) by controlling for gender and age. Table 4 displays the results of these analyses. In Model 1.a, VSWM completely mediated the relationship between EF and mental rotation (direct effect = 0.182, *p* = 0.222, indirect effect = 0.144, *p* < 0.05, *R*^2^ = 0.18). In Model 1.b, no evidence of a significant mediating effect of VSWM was found (direct effect = -0.015, *p* = 0.911, indirect effect = 0.067, *p* = 0.154, *R*^2^ = 0.17). Of the control variables, gender was found to be a significant predictor of mental rotation in both models (see Table 4), indicating that on average, males do significantly better on this test than females. Age was not found to be a significant predictor of mental rotation for either model (see Table 4).

**Table 4:** Results from mediation analysis concerning the mediation relationship among EF, VSWM (CORSIT), and mental rotation after controlling for age and gender.

Name	Path	Estimate	Standard error	*t*_ratio	*p_value*
**Mediation Model 1.a: the Tower Test Indicates EF**					
a	CORSIT ← TOWERA	0.300	0.086	3.490	0.000
b	MRTT ← CORSIT	0.478	0.189	3.440	0.001
**c**	**MRTT** ← **TOWERA**	**0.182**	**0.149**	**1.222**	**0.222**
ag	CORSIT ←GENDER	0.238	0.426	0.558	0.577
aa	CORSIT ← AGE	-0.032	0.033	-0.964	0.335
cg	MRTT ← GENDER	2.266	0.709	3.195	0.001
ca	MRTT ← AGE	-0.086	0.055	-1.542	0.123
s1	TOWERA ↔ TOWERA	4.572	0.541	8.456	0.000
s2	GENDER ↔ GENDER	0.196	0.023	8.456	0.000
s3	AGE ↔ AGE	31.700	3.749	8.456	0.000
cs4	GENDER ↔ TOWERA	0.185	0.080	2.293	0.022
cs5	AGE ↔ GENDER	0.664	0.215	3.082	0.002
cs6	AGE ↔TOWERA	1.911	1.019	1.874	0.061
s7	CORSIT ↔ CORSIT	4.599	0.544	8.456	0.000
s8	MRTT ↔ MRTT	12.701	1.502	8.456	0.000
**a^∗^b**	**Mediation effect**	**0.144**	**0.059**	**2.450**	**0.014**
**Mediation Model 1.b: the Color-Interference Test Indicates EF**					
a	CORSIT ← CITINSW	0.123	0.081	1.526	0.127
b	MRTT ← CORSIT	0.539	0.135	3.995	0.000
**c**	**MRTT** ←**CITINSW**	**-0.015**	**0.132**	**-0.112**	**0.911**
ag	CORSIT ← GENDER	0.455	0.439	1.037	0.300
aa	CORSIT ← AGE	-0.018	0.034	-0.529	0.597
cg	MRTT ← GENDER	2.499	0.711	3.517	0.000
ca	MRTT ← AGE	-0.071	0.055	-1.282	0.200
s1	CITINSW ↔ CITINSW	5.289	0.626	8.456	0.000
s2	GENDER ↔ GENDER	0.191	0.023	8.456	0.000
s3	AGE ↔ AGE	31.088	3.677	8.456	0.000
cs4	GENDER ↔ CITINSW	-0.009	0.084	0.109	0.913
cs5	AGE ↔ GENDER	0.585	0.209	2.795	0.005
cs6	AGE ↔ CITINSW	-0.203	1.072	0.190	0.850
s7	CORSIT ↔ CORSIT	4.947	0.585	8.456	0.000
s8	MRTT ↔ MRTT	12.872	1.522	8.456	0.000
**a^∗^b**	**Mediation Effect**	**0.067**	**0.047**	**1.426**	**0.154**

### Mediation by VWM

The second set of mediation models looked at whether the prediction of mental rotation by EF was mediated by VWM (DIGIT). Again, in Model 2.a, EF was measured by the Tower test and in Model 2.b, EF was measured by the Color-Interference Test. Indirect effects were tested using Sobel test by controlling for gender and age. Table 5 displays the results of these analyses. In neither Model 2.a nor Model 2.b did VWM completely mediate the relationship between EF and mental rotation. Of the control variables, again, gender was found to be a significant predictor of mental rotation in both models (see Table 5), indicating that on average, males do significantly better on this test than females, whereas age was not found to be a significant predictor of mental rotation for either model (see Table 5).

**Table 5:** Results from mediation analysis concerning the mediation relationship among EF, VWM (DIGIT), and mental rotation after controlling for age and gender.

Name	Path	Estimate	Standard error	*t*_ratio	*p_value*
**Mediation Model 2.a: the Tower Test indicates EF**					
a	DIGIT ← TOWERA	0.226	0.098	2.285	0.022
b	MRTT ← DIGIT	0.104	0.125	0.822	0.410
**c**	**MRTT** ← **TOWERA**	**0.302**	**0.151**	**2.000**	**0.045**
ag	DIGIT ← GENDER	0.402	0.488	0.821	0.411
aa	DIGIT ← AGE	0.040	0.038	1.036	0.299
cg	MRTT ← GENDER	2.338	0.737	3.171	0.001
ca	MRTT ← AGE	-0.105	0.057	-1.822	0.068
s1	TOWERA ↔ TOWERA	4.572	0.540	8.455	0.000
s2	GENDER ↔ GENDER	0.196	0.023	8.455	0.000
s3	AGE ↔ AGE	31.703	3.749	8.455	0.000
cs4	GENDER ↔ TOWERA	0.185	0.080	2.292	0.021
cs5	AGE ↔ GENDER	0.664	0.215	3.081	0.002
cs6	AGE ↔ TOWERA	1.911	1.019	1.874	0.060
s7	DIGIT ↔ DIGIT	6.045	0.714	8.455	0.000
s8	MRTT ↔ MRTT	13.687	1.618	8.455	0.000
**a^∗^b**	**Mediation effect**	**0.023**	**0.030**	**0.774**	**0.439**
**Mediation Model 2.b: the Color-Interference Test Indicates EF**					
a	DIGIT ← CITINSW	0.080	0.091	0.873	0.382
b	MRTT ← DIGIT	0.150	0.125	1.192	0.233
**c**	**MRTT** ←**CITINSW**	**0.040**	**0.137**	**0.290**	**0.771**
ag	DIGIT ← GENDER	0.597	0.495	1.205	0.227
aa	DIGIT ← AGE	0.052	0.038	1.340	0.180
cg	MRTT ← GENDER	2.655	0.746	3.556	0.000
ca	MRTT ← AGE	-0.089	0.058	-1.516	0.129
s1	CITINSW ↔ CITINSW	5.289	0.625	8.455	0.000
s2	GENDER ↔ GENDER	0.191	0.022	8.455	0.000
s3	AGE ↔ AGE	31.088	3.676	8.455	0.000
cs4	GENDER ↔ CITINSW	-0.009	0.083	-0.109	0.912
cs5	AGE ↔ GENDER	0.585	0.209	2.794	0.005
cs6	AGE ↔ CITINSW	-0.203	1.072	-0.189	0.849
s7	DIGIT ↔ DIGIT	6.301	0.745	8.455	0.000
s8	MRTT ↔ MRTT	14.168	1.675	8.455	0.000
**a^∗^b**	**Mediation Effect**	**0.012**	**0.017**	**0.705**	**0.481**

### Mediation by VSWM and VWM

In the third set of mediation models, both mediators, VSWM (CORSIT) and VWM (DIGIT), were entered into the model simultaneously in predicting mental rotation. In Model 3.a, VSWM completely mediated the relationship between EF and mental rotation (direct effect = 0.181, *p* = 0.226, indirect effect = 0.144, *p* < 0.05, *R*^2^ = 0.18). In the same model, VWM was not a significant mediator between EF and mental rotation. In Model 3.b, neither VSWM nor VWM was a significant mediator. Of the control variables, gender was found to be a significant predictor of mental rotation in both models (see Table 6), indicating that on average, males do significantly better on this test than females. Age was not found to be a significant predictor of mental rotation for either model (see Table 6).

**Table 6:** Results from mediation analysis concerning the mediation relationship among EF, VSWM (CORSIT), VWM (DIGIT), and mental rotation after controlling for age and gender.

Name	Path	Estimate	Standard error	*t*_ratio	*p_value*
**Mediation Model 3.a: the Tower Test indicates EF**					
a	CORSIT ← TOWERA	0.300	0.086	3.489	0.000
b	MRTT ← CORSIT	0.477	0.143	3.332	0.000
**c**	**MRTT** ← **TOWERA**	**0.181**	**0.150**	**1.209**	**0.226**
ag	CORSIT ← GENDER	0.238	0.426	0.558	0.576
aa	CORSIT ← AGE	-0.032	0.033	-0.964	0.334
cg	MRTT ← GENDER	2.264	0.710	3.187	0.001
ca	MRTT ← AGE	-0.086	0.055	-1.537	0.124
d	DIGIT ← TOWERA	0.226	0.098	2.285	0.022
da	DIGIT ← AGE	0.040	0.038	1.036	0.299
dg	DIGIT ← GENDER	0.402	0.488	0.821	0.411
e	MRTT ← DIGIT	0.005	0.124	0.036	0.970
s1	TOWERA ↔ TOWERA	4.572	0.540	8.455	0.000
s2	GENDER ↔ GENDER	0.196	0.023	8.455	0.000
s3	AGE ↔ AGE	31.703	3.749	8.455	0.000
cs4	GENDER ↔ TOWERA	0.185	0.080	2.292	0.021
cs5	AGE ↔ GENDER	0.664	0.215	3.081	0.002
cs6	AGE ↔ TOWERA	1.911	1.019	1.874	0.060
s7	CORSIT ↔ CORSIT	4.599	0.543	8.455	0.000
s8	MRTT ↔ MRTT	12.700	1.501	8.455	0.000
s9	DIGIT ↔ DIGIT	6.045	0.714	8.455	0.000
cv10	CORSIT ↔ DIGIT	1.254	0.453	2.767	0.005
**a^∗^b**	**Mediation effect**	**0.143**	**0.059**	**2.409**	**0.015**
**d^∗^e**	**Mediation effect**	**0.001**	**0.028**	**0.036**	**0.970**
**Mediation Model 3.b: the Color-Interference Test Indicates EF**					
a	CORSIT ← CITINSW	0.123	0.080	1.526	0.126
b	MRTT ← CORSIT	0.531	0.140	3.798	0.000
**c**	**MRTT** ←**CITINSW**	**-0.015**	**0.131**	**-0.119**	**0.904**
ag	CORSIT ← GENDER	0.455	0.438	1.037	0.299
aa	CORSIT ← AGE	-0.018	0.034	-0.529	0.596
cg	MRTT ← GENDER	2.488	0.712	3.491	0.000
ca	MRTT ← AGE	-0.072	0.055	-1.295	0.195
d	DIGIT ← CITINSW	0.079	0.091	0.873	0.382
da	DIGIT ← AGE	0.052	0.038	1.340	0.180
dg	DIGIT ← GENDER	0.597	0.495	1.205	0.227
e	MRTT ← DIGIT	0.023	0.124	0.186	0.852
s1	CITINSW ↔ CITINSW	5.289	0.625	8.455	0.000
s2	GENDER ↔ GENDER	0.190	0.022	8.455	0.000
s3	AGE ↔ AGE	31.088	3.676	8.455	0.000
cs4	GENDER ↔ CITINSW	-0.009	0.083	-0.109	0.912
cs5	AGE ↔ GENDER	0.585	0.209	2.794	0.005
cs6	AGE ↔ CITINSW	-0.203	1.072	-0.189	0.849
s7	CORSIT ↔ CORSIT	4.947	0.585	8.455	0.000
s8	MRTT ↔ MRTT	12.868	1.521	8.455	0.000
s9	DIGIT ↔ DIGIT	6.301	0.745	8.455	0.000
cv10	CORSIT ↔ DIGIT	1.497	0.483	3.098	0.001
**a^∗^b**	**Mediation effect**	**0.066**	**0.046**	**1.416**	**0.156**
**d^∗^e**	**Mediation effect**	**0.002**	**0.010**	**0.182**	**0.855**

### Dominance Analysis

The main conclusions that can be drawn from the dominance analyses are: (1) VSWM is more important than VWM in explaining individual differences in performance on the mental rotation test, as reflected by the *R*^2^ values in multiple regression analyses (0.108 vs. 0.014). (2) The total achievement score on the Tower test (TOWERA) is more important than the composite inhibition and switching score on the Color-Interference Test (CINITSW), as indicated by the *R*^2^ values (0.041 vs. 0.001). (3) Lastly, gender is more important than age in explaining the variance of the outcome variable, mental rotation, as reflected by the *R*^2^ values (0.075 vs. 0.002).

## Discussion

To explore whether a test effect exists, we investigated whether using EF tests that have different secondary demands makes a difference in the mediation relationship tested, as well as in predicting individual differences in spatial ability. The present study was motivated by Miyake et al. (2001) and Kaufman (2007) studies. One goal of the study was to investigate the relationships among EF, VSWM, VWM, and mental rotation by testing a series of mediation models that explored the following research questions: (1)Whether VSWM mediates the relationship between EF and mental rotation when EF is measured by the Tower test, which has a strong visuospatial secondary demand (i.e., Model 1a); (2)Whether VSWM mediates the relationship between EF and mental rotation when EF is measured by the Color-Interference Test, which has a strong verbal secondary demand (i.e., Model 1b); (3)Whether VWM mediates the relationship between EF and mental rotation, when EF is measured by the Tower test, which has a strong visuospatial secondary demand (i.e., Model 2a); (4)Whether VWM mediates the relationship between EF and mental rotation, when EF is measured by the Color-Interference Test, which has a strong verbal secondary demand (i.e., Model 2b); (5) Whether VSWM or VWM significantly mediates the relationship between EF and mental rotation, when both mediation relationships are simultaneously tested in the same model and when EF is measured by the Tower test, which has a strong visuospatial secondary demand (i.e., Model 3a); (6)Whether VSWM or VWM significantly mediates the relationship between EF and mental rotation, when both mediation relationships are simultaneously tested in the same model and when EF is measured by the Color-Interference Test, which has a strong verbal secondary demand (i.e., Model 3b).

The results of model testing suggest that when the Tower test, an EF test with a strong spatial processing demand, was used to represent EF, VSWM significantly mediated the relationship between EF and mental rotation but when the combined switching and inhibition scores from the Color-Interference Test, an EF test with a strong verbal demand, was used to represent EF, VSWM no longer significantly mediated the relationship between EF and mental rotation. The results hold when both VSWM and VWM serve as the mediators in the model. This pattern of findings suggests that the test effect exists. Therefore, cautions need to be taken when interpreting the significance of EF’s impact on VSWM and/or spatial ability from previously published studies that used a variety of EF measures, some of which have a strong spatial secondary demand, whereas others have a strong verbal secondary demand. Furthermore, future studies investigating the relationship between EF and VSWM or between EF and spatial ability need to find a way to actively control for the confounding influence of the secondary demand inherent in the EF tests used. This is especially important if the objective of the study is to understand the implication of the task-pure EF processes in VSWM and spatial ability. In regard to VWM, the results of the mediation analyses tested in Models 2a, 2b, 3a, and 3b did not find VWM to mediate the relationship between EF and mental rotation at a statistically significant level, regardless of whether the Tower test or the Color-Interference Test was used to represent the EF.

The present study is an extension to Miyake et al. (2001) study in that those authors examined the relationship between EF and spatial ability and between EF and VSWM in separate models, without investigating mediation relationships. The present study fills this gap in knowledge. Secondly, in our review of the working memory literature, it can be inferred that VSWM and mental rotation are similar theoretical constructs. EF can be further divided into inhibition, shifting, and updating components. The updating component of the EF overlaps with VSWM and VWM at a functional level in the sense that it fulfills the role of temporarily storing and updating incoming information, regardless of its modality. Thus, if it were the shared variance between the updating component of the EF, VSWM, and VWM that contributed to individual differences in spatial ability (mental rotation), rather than the shared spatial demand inherent in some tests of EF such as the Tower test used in this study and VSWM, one might expect the present study to show that both VSWM and VWM significantly mediate the relationship between EF and mental rotation. However, that was not the case, as revealed by the results of model testing featured in this study, since only VSWM but not VWM significantly mediated the relationship between EF, when measured by the Tower Test, an EF task with a strong spatial secondary demand, and spatial ability in models where VSWM and VWM served as mediators. Based on this pattern of findings, it may be inferred that it is the shared spatial secondary demand between the Tower Test and VSWM that may have accounted for individual differences in mental rotation. Furthermore, the results that VSWM completely mediated the relationship between the Tower Test and mental rotation but not between the Color-Interference Test and mental rotation are consistent with this interpretation. Thirdly, the present study also extended Kaufman (2007) study in that Kaufman (2007) did not include EF as a predictor of mental rotation in the mediation models tested. In this study, EF was included in all the mediational models tested. While Kaufman (2007) study found a significant relationship between VWM and spatial ability, the present study found no significant relationship between the two constructs when EF was also included in the model as a predictor of mental rotation. Nor was VWM found to significantly mediate the relationship between EF and mental rotation, regardless of whether the Tower test or the Color-Interference Test was used to represent the EF (see Tables 5, 6). Fourthly, Models 3.a and 3.b. in the present study combined the analyses performed by Miyake et al. (2001) and Kaufman (2007) by including EF, VSWM, VWM, and spatial ability (mental rotation) in the same model and simultaneously tested two mediation relationships, one involves VSWM as the mediator and the other involves VWM as the mediator, after controlling for the covariates age and gender. In Model 3.a, when EF is indicated by the Tower Test, VSWM completely mediated the relationship between EF and mental rotation, whereas VWM is not a significant mediator. In Model 3.b, when EF is indicated by the Color-Interference Test, neither VSWM nor VWM significantly mediated the relationship between the EF and mental rotation. Lastly, regarding the two covariates, in all models tested, gender had a significant impact on the mediation relationship, whereas age did not. Specifically, being a male is associated with better mental rotation ability. This gender effect is not surprising and is consistent with the well-documented male advantage in spatial ability reported in the gender differences literature (see Linn and Petersen, 1985; Voyer et al., 1995). In addition, being a male was found to be associated with better performance on the Tower Test, which is also not surprising, given that the Tower Test has a strong spatial secondary demand.

Another objective of the study is to investigate the relative importance of EF, VSWM, and VWM in predicting mental rotation through dominance analysis. The pattern of findings resulting from these analyses is consistent with the literature. Relative to VWM, VSWM is more powerful in explaining individual differences in mental rotation, most likely due to a shared cognitive demand between the spatial span task and the mental rotation test at the measurement level or due to the similarity between VSWM and spatial ability (and in particular, mental rotation) at the construct level. The finding that the Tower Test is more important than the Color-Interference Test in explaining individual differences in mental rotation supplements the results from the mediation analyses and again suggests that the significant relationship between EF and spatial ability reported in prior studies may be due to the spatial secondary demand inherent in some of the EF tests used in those studies. Therefore, it remains to be determined whether at the construct level, EF and spatial ability are as closely related as the previous results appear to indicate.

### Limitations and Future Directions

A limitation of the present study is that the sample is not gender-balanced. While every effort was made to recruit a gender balanced sample, we ended up with more females than males in the working sample. Because our primary research questions do not concern gender differences, and furthermore, in the analyses performed, the gender variable was controlled for, gender imbalance has not been an issue in this study. However, in future studies that aim at comparing gender differences in the magnitude of the mediation relationships tested in this study, acquiring a gender-balanced sample may be advisable.

A second limitation of the present study is its sample size. Ideally, studies featuring mediation analyses should aim for a sample that is sufficiently powered to detect an effect size of 0.8. However, reported that the median of the sample sizes reported in published studies that aimed at detecting indirect mediation effects using a regression approach is 142.5, which is similar to the present sample size of 144.

Finally, in this study, we used the composite score of inhibition and switching on the CIT and the total achievement score on the Tower test to represent EF in the mediation models tested, as well as in the ensuing dominance analysis. The total achievement score on the Tower test tapped the inhibition and updating components of the EF. The analyses performed did not involve identifying the unique effects of inhibition, switching, and updating components of the EF. This is because the primary goal of the present study is to understand the relationships among EF, VSWM, VWM, and mental rotation spatial ability at the construct level, and whether the nature of the secondary task demand in EF (i.e., verbal demand in the CIT and spatial demand in the Tower test) makes a difference in the mediation effects. In future studies, it may be of interest to isolate inhibition, switching, and updating components of the EF and test the mediation relationships separately for each component of the EF. In the present study, we did not isolate those individual components of the EF in order to preserve the psychometric integrity of the tests.

## Datasets are Available on Request

The raw data supporting the conclusions of this manuscript will be made available by the corresponding author, without undue reservation, to any qualified researcher.

## Ethics Statement

This study was carried out in accordance with the recommendations of the guidelines concerning behavioral studies involving human subjects, Institutional Review Board (IRB) at the University of Georgia. The protocol was approved by the Institutional Review Board at the University of Georgia. All subjects gave written informed consent in accordance with the Declaration of Helsinki.

## Author Contributions

LW conducted the research, drafted the Introduction, Materials and Methods, and the Discussion sections. JB participated in the writing of the Results section and revised the Materials and Methods and Results sections. ZL created the figures and tables and participated in the writing of the Results section. MC participated in the design of the study, as well as proofreading earlier drafts of this paper.

## Conflict of Interest Statement

The authors declare that the research was conducted in the absence of any commercial or financial relationships that could be construed as a potential conflict of interest.

## Footnotes

1 Forward and Backward Digit Span tests are part of the Wechsler Memory Scale III and are frequently used in clinical and neuropsychological research. The test manual reports the Cronbach’s α for the Backward Digit Span task to be 0.84. No reliability information was reported for the Forward Digit Span in the test manual but it is reasonable to infer that the Cronbach’s α for the Forward Digit Span will be higher than that of the Backward Digit Span. This is because Forward Digit Span has fewer cognitive demand than Backward Digit Span and therefore performance across items is more likely to be homogenous than the latter. We have good reasons to believe that the Cronbach’s α of the Backward Digit Span task in the current sample is in the same ballpark as reported in the test manual, given the similarity of the present sample to the normed sample.

2 No reliability information was reported for either the Forward or the Backward Spatial Span in the test manual. Forward and Backward Spatial Span tests are part of the Wechsler Memory Scale III and are commonly used in clinical and neuropsychological research.

3 The test manual reports that the internal consistency (split-half reliability coefficients corrected by the Spearman-Brown formula) of the CIT is between 0.62 and 0.86 across different age groups (Homack et al., 2005). There is good reason to believe that the reliability coefficients of the present sample is in the same ballpark as the range reported in the test manual concerning this age group.

4 The test manual reports that the internal consistency of the Tower test ranges from 0.50 to 0.80 across different age groups.

5 The MRT has a split-half reliability of 0.86 when administered to undergraduate students under a 6-min constraint using the Spearman-Brown formula (Qubeck, 1997). When administered to 3,268 adults and adolescents ≥ 14 years of age under a 10-min constraint, applying the Kuder-Richardson formula 20, the internal consistency of the MRT is 0.88 (Wilson et al., 1975). Using a similar sample of 336 participants, the test–retest reliability of the MRT was 0.83 (Vandenberg and Kuse, 1978). Because our sample and testing conditions are very similar to the cited studies, there is good reason to believe that the reliability of the MRT in our sample is within the range reported in the cited studies.
